# A Randomized Controlled Trial of Inhibitory Control Training for the Reduction of Alcohol Consumption in Problem Drinkers

**DOI:** 10.1037/ccp0000312

**Published:** 2018-12

**Authors:** Andrew Jones, Elly McGrath, Eric Robinson, Katrijn Houben, Chantal Nederkoorn, Matt Field

**Affiliations:** 1 Department of Psychological Sciences, University of Liverpool, and UK Centre for Tobacco and Alcohol Studies, Liverpool, United Kingdom; 2 Faculty of Psychology and Neuroscience, Maastricht University; 3 Department of Psychological Sciences, University of Liverpool, and UK Centre for Tobacco and Alcohol Studies, Liverpool, United Kingdom

**Keywords:** alcohol, E-health intervention, inhibitory control training

## Abstract

***Objective:*** We conducted a randomized controlled trial to compare the effects of three types of Internet-delivered Inhibitory Control Training (ICT) with each other and with an active control intervention on alcohol consumption in a community sample of problem drinkers. ***Method:*** Two hundred and 46 heavy drinkers, who were motivated to reduce their alcohol consumption (mean age 41.32, 130 female) self-monitored their alcohol consumption for 1 week before being randomized to receive 1 of 3 variants of ICT (Associative No-Go, Associative Stop Signal, General Inhibition) or an active control. Participants then completed up to 14 ICT/control sessions on the Internet over a 4-week period, while regularly recording their alcohol consumption. ***Results:*** There were significant reductions in alcohol consumption across all groups over the 4-week training period (main effect of time, *F*(2, 402) = 77.12, *p* < .01, η_*p*_^2^ = .28, BF^10^ > 99), however there were no differences between ICT groups, or between ICT groups and the active control group (Group × Time interaction, *F*(6, 402) = 1.10, *p* = .36, η_*p*_^2^ = .02, BF^10^ = 0.03). Contrary to hypotheses, there were no changes in general inhibitory control, the disinhibiting effects of alcohol cues, or alcohol affective associations after ICT. ***Conclusions:*** In this study, which attempted to translate findings from proof-of-concept laboratory studies into a viable behavior change intervention, we found that multiple sessions of ICT delivered over the Internet did not help heavy drinkers to reduce their alcohol consumption beyond nonspecific effects associated with taking part in a trial.

## What is the public health significance of this article?

Findings from this randomized controlled trial demonstrated that Inhibitory Control Training (ICT), a novel Internet-delivered behavioral intervention that is intended to improve inhibitory control, did not help problem drinkers to reduce their alcohol consumption beyond the nonspecific effects associated with taking part in a trial and regular self-monitoring of alcohol consumption. These findings suggest that interventions developed in laboratory settings may require substantial modification if they are to be translated info effective behavior change interventions suited for remote delivery.

Inhibitory Control Training (ICT) refers to a broad class of computerized behavioral interventions that have the goal to train participants to either improve their general capacity for inhibitory control or to develop associations between alcohol-related cues and engagement of inhibitory control. A number of laboratory studies have demonstrated that single sessions of ICT can reduce alcohol consumption in bogus “taste tests” (see meta-analyses by Allom, Mullan, & Hagger, 2016; Jones, Di Lemma et al., 2016). Furthermore, there is emerging evidence that multiple sessions of food-related ICT delivered via the Internet can help people to reduce unhealthy food intake and lose weight (Allom & Mullan, 2015; Lawrence et al., 2015; Veling, van Koningsbruggen, Aarts, & Stroebe, 2014). To date, there is no evidence that the promising findings for alcohol ICT from laboratory studies can be translated into an efficacious behavior change intervention for the reduction of alcohol consumption. In the present article we report findings from a randomized controlled trial in which we compared three conceptually distinct types of ICT with each other and with an active control for their sustained effects on alcohol consumption in a community sample of problem drinkers.

Inhibitory control is defined as the ability to stop, change, or delay an inappropriate response (Logan, Cowan, & Davis, 1984), and it is a key component of broader constructs such as executive functioning, impulsivity, and self-control (Bickel, Jarmolowicz, Mueller, Gatchalian, & McClure, 2012; Fujita, 2011; Miyake et al., 2000). Inhibitory control can be assessed with computerized tasks such as the Stop Signal and Go/No-Go tasks. These tasks establish prepotent rapid motor responding to visual cues, but on a minority of trials a Stop Signal or No-Go cue is presented which indicates to participants that they should refrain from responding on that trial. ICT was developed on the basis of observations that alcohol use disorders (AUDs) are associated with deficits in inhibitory control. Alcohol-dependent patients and heavy drinkers recruited from college campuses and community settings tend to perform poorly on the aforementioned computerized tasks, relative to light drinking or abstainer controls (Christiansen, Cole, Goudie, & Field, 2012; Houston et al., 2014; Lawrence, Luty, Bogdan, Sahakian, & Clark, 2009; Smith, Mattick, Jamadar, & Iredale, 2014). Furthermore, deficits in inhibitory control are exacerbated during exposure to alcohol-related cues, in both dependent (Czapla et al., 2016; Gauggel et al., 2010) and nondependent drinkers (Jones & Field, 2015; Petit, Kornreich, Noël, Verbanck, & Campanella, 2012; Weafer & Fillmore, 2012, 2015). We recently demonstrated that these disinhibiting effects of alcohol cues partially mediate subsequent alcohol consumption in heavy drinkers who are attempting to reduce their consumption (Field & Jones, 2017). This evidence is consistent with claims that poor inhibitory control plays a causal role in the onset of drinking episodes and in the development and maintenance of AUDs (De Wit, 2009; Goldstein & Volkow, 2011).

Importantly, it may be possible to “train” inhibitory control, either by improving the general capacity for inhibition through repeated practice of inhibition or other “self-control” tasks with escalating difficulty (general ICT; see Friese, Frankenbach, Job, & Loschelder, 2017), or by using associative learning principles to directly pair alcohol-related cues with engagement of inhibitory control, so that those cues automatically evoke inhibition when they are subsequently encountered (associative ICT; see Jones, Hardman, Lawrence, & Field, 2018; Stice, Lawrence, Kemps, & Veling, 2016). Regarding general ICT, findings from proof-of-concept laboratory studies demonstrate that changing the wording of task instructions in order to place the emphasis on successful inhibition (rather than rapid responding) during the Stop Signal task can lead to short-lived improvements in inhibitory control that are accompanied by reductions in alcohol consumption (Jones, Cole, Goudie, & Field, 2011; Jones, Guerrieri et al., 2011). Regarding associative ICT, Houben, Nederkoorn, Wiers, and Jansen (2011) embedded alcohol-related and control pictures into a Go/No-Go task and instructed participants to respond (Go) or refrain from responding (No-Go) in response to letters that appeared in the corner of those pictures. In the ICT group, the signal to respond was consistently paired with control pictures, whereas the signal to inhibit was consistently paired with alcohol pictures; these contingencies were reversed in a control group. Primary findings were reduced self-reported alcohol consumption over the subsequent week in the ICT group compared to the control group, and these effects were mediated by changes in automatic affective responses to alcohol-related pictures. In a subsequent study, Jones and Field (2013) embedded alcohol-related cues into a Stop Signal task and paired alcohol-related pictures with the occurrence of stop signals in the ICT group. Immediately after completion of a single session of ICT, the ICT group consumed less alcohol in the laboratory compared to control groups in which stop signals were paired with neutral pictures or which did not inhibit responding at all. Two recent meta-analyses that incorporated these and other laboratory studies demonstrated a robust, small to moderate (*d ∼* .43) effect of a single session of associative ICT on alcohol consumption, compared to control interventions (Allom et al., 2016; Jones et al., 2016; see also Di Lemma & Field, 2017; Smith, Dash, Johnstone, Houben, & Field, 2017).

Few studies have investigated if a single session of ICT could influence alcohol consumption outside of the laboratory, or elicit a sustained reduction in drinking, and findings from these studies have been contradictory (see Allom et al., 2016). However, completion of multiple sessions of food-related associative ICT outside of the laboratory leads to robust reductions in snack intake and accompanying weight loss in disinhibited eaters or people who are dieting to lose weight (Allom & Mullan, 2015, Study 1; Lawrence et al., 2015; Veling et al., 2014). In each of these studies ICT was administered online on multiple occasions: four sessions in 1 week (Lawrence et al., 2015), one session per week for 4 weeks (Veling et al., 2014), or one session per day for 10 days (Allom & Mullan, 2015, Study 1). All studies compared associative ICT to control groups who did not inhibit to food-related cues. Each of these studies reported a significant reduction in body mass index after ICT compared to control interventions (e.g., *d* = 0.57 in Lawrence et al., 2015); however, see Allom and Mullan (2015, Study 2) for a failure to replicate. Similarly, a mobile phone-based game that incorporated components of ICT (repeated inhibition to unhealthy food-related cues) alongside rapid approach to healthy food cues demonstrated beneficial effects on food intake (Blackburne, Rodriguez, & Johnstone, 2016). However due to the design of this study and the choice of control group (wait-list, as opposed to an active control) these findings are difficult to interpret (see discussion in Jones et al., 2018).

To date, only one study has investigated the effects of repeated sessions of general ICT on alcohol consumption outside the laboratory. Bartsch, Kothe, Allom, Mullan, and Houben (2016) instructed their participants (students who were not motivated to reduce their alcohol consumption) to complete a Stop Signal task every day for 4 consecutive days. A control group repeatedly completed a simple speeded categorization task with no requirement to inhibit. They demonstrated no group differences in alcohol consumption at follow-up 4 weeks later. Although, at face value, this study suggests that general ICT has no sustained effects on alcohol consumption, it is notable that the study population were not motivated to reduce their alcohol consumption, and one would not expect ICT to exert a prolonged influence on health-related behaviors unless recipients are motivated to change their behavior (see Friese et al., 2017).

The primary aim of the present study was to investigate the effects of different types of ICT on alcohol consumption in problem drinkers who were motivated to reduce their drinking. We developed and tested three distinct forms of ICT on the basis of the existing literature. First, Associative No-Go ICT, in which there was a 100% contingency between alcohol and the requirement to inhibit in the context of a modified Go/No-Go task. Second, Associative Stop-Signal ICT, in which there was a 50% contingency between alcohol and the requirement to inhibit in the context of a modified Stop Signal task. Third, general Stop-Signal ICT, in which participants performed a modified Stop Signal task that escalated in difficulty over time, but participants did not learn to associate alcohol cues with the requirement to inhibit.^1^ ICT recruits subtly different forms of inhibitory control depending on the type of task: in Associative No-Go training, the cue to inhibit (“No Go” cue) and alcohol pictures are presented simultaneously, therefore *action restraint* is trained. Whereas in Associative Stop Signal training, there is a brief delay between presentation of the alcohol picture and the cue to inhibit (“Stop Signal”), therefore *action cancellation* is trained (see Eagle, Bari, & Robbins, 2008).

We recruited problem drinkers (adults who self-reported drinking alcohol in excess of U.K. government guidelines for low-risk drinking) who were motivated to reduce their alcohol consumption, from the local community. This population of problem drinkers constitute a significant public health concern in the U.K. and many other countries (Rehm et al., 2009), in part because they are unlikely to seek help and because if they do seek help, the efficacy, acceptability and implementation of current interventions for this population is inadequate (Barry et al., 2004; Kaner et al., 2013). Importantly, E-Health interventions have been identified as a promising alternative to existing interventions for this population (Riper et al., 2011). We recruited participants who self-reported being motivated to reduce their alcohol consumption because motivation to change behavior is a prerequisite for engagement of inhibitory control in tempting situations in order to facilitate longer-term goals such as the reduction of alcohol intake (Hofmann, Friese, & Strack, 2009; Hofmann, Schmeichel, & Baddeley, 2012), and also because motivation to change is likely to maximize compliance with treatment protocols (Jin, Sklar, Oh, & Li, 2008). We encouraged participants to complete up to 14 sessions of ICT over a 28-day period, which is the largest number of ICT sessions studied to date. We also investigated the mechanisms of action of different forms of ICT. Specifically, we anticipated that general ICT would improve inhibitory control capacity, whereas Associative Stop signal ICT would dampen the disinhibiting effects of alcohol cues. We also hypothesized that Associative No-Go ICT would devalue alcohol-related cues because repeated and consistent suppression of motor behavior during exposure to appetitive stimuli causes a response conflict that is resolved through devaluation of those cues (Chen, Veling, Dijksterhuis, & Holland, 2016; Veling, Aarts, & Stroebe, 2013; Veling, Holland, & van Knippenberg, 2008), and these devaluation effects may mediate the effects of ICT on alcohol consumption (Houben et al., 2011). On the basis of previous studies that demonstrated these devaluation effects for alcohol cues after ICT at the time the present study was planned (Houben, Havermans, Nederkoorn, & Jansen, 2012; Houben et al., 2011), we used an alcohol-related implicit association test (IAT) to monitor the hypothesized devaluation effects.

Our primary hypotheses were:

*Hypothesis 1:* All ICT groups would report reductions in alcohol consumption, compared to the control group, during training and at follow-up. We had no specific predictions about which type of ICT would be most effective.

*Hypothesis 2:* ICT would lead to changes in candidate mechanisms of action, which would differ by type of ICT. Specifically, general ICT would lead to improvements in inhibitory control capacity, Associative No-Go ICT would cause devaluation of alcohol-related cues (as inferred from performance on the IAT), and Associative Stop Signal ICT would reduce the disinhibiting effects of alcohol-related cues (as inferred from performance on a cued Stop Signal task).

We also conducted a number of additional exploratory analyses that were not preregistered. These examined compliance and the “dose” of ICT received (the number of sessions completed), and individual differences in changes in the disinhibiting effects of alcohol cues after ICT, as factors that should be associated with the effects of ICT on alcohol consumption (see Jones et al., 2016). This RCT was preregistered (Trial Registration: ISRCTN55671858) and the methodology, primary hypotheses, and analysis strategy were published before data collection commenced (Jones et al., 2014).

## Method

### Trial Design

We conducted a randomized controlled trial with four parallel groups. Participants were randomized using a random number generator with no additional stratification, to ensure unbiased randomization (Suresh, 2011). There were no changes to the methods described in the study protocol (Jones et al., 2014) before or during recruitment of participants.

### Participants

A total of 246 (130 Male) participants with a mean age of 41.32 ± 11.74 years were recruited into the study. This was larger than the intended sample size for our primary analyses (179; see Jones et al., 2014), but smaller than required to exclude the 33% of the sample (*N* = 268) that we anticipated would reduce their alcohol intake after 1 week of self-monitoring but before randomization to intervention arms (this secondary analysis of “reducers” is presented in online supplementary materials).^2^ The study was advertised via university intranet, media (radio and newspaper), and workplace leaflets in Merseyside, U.K. Inclusion criteria were age 25–65, self-reported drinking in excess of U.K. government guidelines for low-risk drinking (>21 units for males, >14 units for females^3^; Edwards, 1996), and self-reported motivation to reduce alcohol consumption. Exclusion criteria were any history of treatment for an AUD, or a current or previous diagnosis of substance use disorder (including AUD), and/or attention deficit disorder. We specifically did not recruit individuals who had been diagnosed with or received treatment for AUD for ethical reasons: repeated exposure to alcohol-related cues during ICT may have increased the risk of (re)lapse (Carter & Tiffany, 1999; Tiffany & Carter, 1998). Participants also had to have access to a computer with a keyboard (desktop or laptop) and Internet access. The project was approved by the University of Liverpool’s Research Ethics Committee.

### Pictorial Stimuli Used in Laboratory Assessments and Online ICT Sessions

For the cue-specific Stop Signal (assessment) task, Associative Stop Signal and No-Go ICT, and control (training) tasks we used 10 alcohol and matched stationery pictures that have been used in our previous studies (e.g., Jones & Field, 2013). Each alcohol picture had a matched stationery picture (e.g., a man with a glass of lager to his lips, a man with a pen to his lips), and picture pairs were matched closely for composition, complexity and brightness. A variety of different beverage types were presented (beer, wine, cider, spirits) and a subset of these images were used in the IAT (assessment).

### Laboratory Assessment Tasks (for Detailed Descriptions, see Jones et al., 2014)

#### Stop Signal task (Verbruggen, Logan, & Stevens, 2008)

Each trial began with a fixation cross (+) presented in the center of the computer screen for 500 ms. This was followed by a left or right facing arrow (the Go stimulus). Participants were instructed to make a speeded categorization response to the direction of this arrow by pressing one of two keys on the keyboard. On 75% of all trials this was uninterrupted (Go trials). On the remaining 25% of trials a short auditory tone (the Stop Signal) occurred after the Go stimulus, and participants were instructed to inhibit their categorization response if they heard the tone (Stop Trials). The delay between the Go stimulus and Stop Signal onset (the Stop Signal Delay, SSD) was initially set at 250 ms. If participants successfully inhibited a response, the delay on the subsequent stop trial increased by 50 ms (making inhibition more difficult), and if the participant failed to inhibit, the delay decreased by 50 ms (making inhibition easier). The minimum SSD was 0 ms (i.e., the Stop Signal could not appear before the Go stimulus), and the maximum SSD was 1,250 ms. Participants completed a practice block of 16 trials (4 stop trials), followed by three experimental blocks of 64 trials (16 stop trials).

#### Cue-specific Stop Signal task

The cue-specific Stop Signal task was similar to the standard Stop Signal task, except that participants had to categorize alcohol-related pictures by pressing one key if the picture was presented in portrait format and a different key if the picture was presented in landscape format. Participants completed a practice block of 16 trials (4 stop trials), followed by three experimental blocks of 64 trials (16 stop trials). Each Stop Signal task took 10–12 min to complete.

#### Alcohol Valence Implicit Association Task (IAT, see Houben et al., 2012)

The IAT required participants to classify pictures and words into two target categories (alcohol and stationery pictures) and two affective categories (positive and negative words) as quickly as possible. Positive words (*happy*, *jolly*, *energetic*, *funny*, *sociable*, *cheerful*) and negative words (*dull*, *miserable, sick, depressed*, *unhappy*, *drowsy*) were those used in Houben et al. (2012). The IAT consisted of seven blocks. In Blocks 1 (24 trials) and 2 (24 trials) participants categorized the pictorial targets and affective words, respectively. The next blocks were combination blocks: In Block 3 (practice; 24 trials) and Block 4 (test; 48 trials) participants categorized all four pictorial target and affective category words, with one pictorial target and one affective category assigned to one key, and the other pictorial target and the other affective category assigned to the other key (e.g., positive words and alcohol pictures were mapped to one key, whereas negative words and stationery pictures were mapped to the other key). In Block 5 (48 trials) the keys for target classification were reversed; this block required categorization of the alcohol and stationery pictures only. The remaining blocks were combination blocks: In Block 6 (practice; 24 trials) and Block 7 (test; 48 trials) participants again categorized all four pictorial target and affective category words using only two response keys, but this time the stimulus pairings were reversed from those applied in Blocks 3 and 4 (e.g., positive words and stationery pictures were mapped to one key, whereas negative words and alcohol pictures were mapped to the other key). Response key assignment and order of combination blocks (3 and 4; 6 and 7) were counterbalanced across participants. There were 240 trials in total and the task took 8–10 min to complete.

### Training Tasks Delivered Online (for Detailed Descriptions see Jones et al., 2014)

#### Associative Stop-Signal training

Participants were instructed to categorize centrally presented alcohol and stationery pictures based on their content (alcohol or stationery) as quickly as possible by pressing one of two keys on the keyboard. On Stop trials, the Stop Signal (two horizontal red lines =) was superimposed over the image, and participants were instructed to inhibit their categorization response. The Stop Signal was presented on 50% of alcohol trials and 0% of stationery trials. There were 200 trials in total: 100 Go stationery trials, 50 Go alcohol trials and 50 Stop alcohol trials, with a short break after 100 trials.

#### General inhibition training

Participants rapidly categorized arbitrary stimuli (X or O) by pressing one of two keys. The stop signal (two horizontal red lines =) was superimposed over the X on 50% of trials but was never presented over the O. There were 200 trials in total: 100 Go O trials, 50 Go X trials and 50 stop X trials, with a short break after 100 trials.

The Associative Stop Signal and general inhibition training tasks were similar in that the Stop Signal Delay (SSD) was fixed during each session. In the first session the SSD was always 250 ms. If participants successfully inhibited on at least 50% of stop trials, the SSD increased by 10 ms in the following training session (therefore inhibition was more difficult). If they did not manage to inhibit on at least 50% of the stop trials, the SSD remained the same in the next training session. Therefore if participants completed every training session and successfully inhibited on at least 50% of stop trials, their final SSD would be 380 ms [250 ms + (13 × 10 ms)].

#### Associative No-Go training

Participants were required to rapidly identify and respond (or not respond) to letters that were presented in the corners of alcohol or stationery pictures. Participants were instructed to respond quickly by pressing the space bar if the letter *P* was presented, but to withhold their response if the letter *R* was presented. The letter *R* was always presented in the corner of alcohol images. There were 200 trials (100 alcohol and 100 neutral), with a short break after 100 trials.

We chose to apply a 100% contingency between alcohol pictures and No-Go signals because this is commonly applied in laboratory studies (Houben et al., 2011). Although this meant that Associative No-Go and Stop-Signal ICT sessions differed in terms of the number of inhibition trials (100 vs. 50) and the alcohol-inhibition contingency (100 vs. 50%), this method ensured that interventions were matched in terms of exposure to alcohol-related cues, and that inhibitory “pressure” was maximized in Stop-Signal ICT by making the requirement to inhibit relatively infrequent (see Verbruggen & Logan, 2008).

#### Active control

The control group rapidly categorized alcohol and stationery pictures without any requirement for inhibition. There were 200 trials (100 alcohol and 100 stationery), with a short break after 100 trials.

### Procedure

Participants registered their initial interest in the study by responding to an advertisement via e-mail or telephone; in response, they were sent a participant information sheet by e-mail. The advertisement stated that only “individuals who are motivated to reduce their drinking, and would be willing to try to ‘cut down’” should volunteer to participate, and this was reaffirmed during initial contact. Three male respondents who self-reported a current diagnosis of AUD were excluded from participation at this stage. At least 48 hr after receiving the information sheet, participants were invited to attend the initial laboratory visit at the University of Liverpool. During this visit the study was explained to the participant and they provided informed consent. Following this, they completed a 2-week retrospective recall diary of alcohol consumption (Timeline Follow-Back [TLFB]; Sobell & Sobell, 1992; Cronbach’s alpha = .79), the Alcohol Use Disorders Identification Test (AUDIT; Babor, Higgins-Biddle, Saunders, & Monteiro, 2001; α = .73) to assess hazardous drinking, the Temptation and Restraint Inventory (TRI; Collins & Lapp, 1992) to assess drinking restraint, (Cognitive Behavioral Control subscale; α = .79) and preoccupation with alcohol (Cognitive Emotional Preoccupation subscale; α = .88), and the Barratt Impulsivity Scale (BIS; Patton, Stanford, & Barratt, 1995) to assess self-reported motor (α = .58), attentional (α = .12), and nonplanning impulsivity (α = .28). All participants then completed an online alcohol intervention (Down Your Drink; Linke, Brown, & Wallace, 2004) in order to increase their motivation to reduce their alcohol consumption. Under the supervision and guidance of the researcher, participants created an account and completed the “Quick Visit” option, which provided feedback based on drinking habits, information about health risks, and prompted participants to set a goal for reduction of drinking. This intervention has demonstrable efficacy for increasing motivation to reduce drinking and reducing the volume of alcohol consumed (Wallace et al., 2011). We did not aim to evaluate the effectiveness of Down Your Drink, but we incorporated it in order to increase participants’ motivation to reduce their alcohol intake before and during the training period. Upon completion of the Quick Visit section on Down Your Drink, participants were instructed to keep a detailed daily drinking diary for the remainder of the study, by logging in to the website every day.

Participants returned to the laboratory 1 week later, reaffirmed their consent, and reported their alcohol consumption during this period (on the basis of daily drinking data which they obtained by logging in to their Down Your Drink account). There was a significant reduction in alcohol consumption during this week of self-monitoring, as described in the online supplementary materials. Following this, participants completed the computerized tasks (SST, cue-specific SST, IAT; counterbalanced), which took approximately 30 min in total. They were then given information about the online ICT intervention and provided an e-mail address to which links for individual training sessions could be sent. Participants then left the laboratory before being randomized to one of the four experimental groups. Approximately every other day over the 28-day training period (see below), participants were sent a personalized e-mail from a study-specific e-mail account that contained a link to their training task for that day. The e-mail also contained their unique participant identification number and a reminder to complete their drinking diary on the Down Your Drink website. The links contained in the e-mails directed participants to a study site hosted on Inquisit web (Millisecond Software, Seattle, WA), which initially prompted them to enter their participant number before estimating the number of units of alcohol that they had consumed since their previous contact (which was either the laboratory visit, or the previous training session). Following this, the training task began. Participants were encouraged to complete up to 14 sessions over the 28-day training period (i.e., one session every other day). Initially, data from each participant was checked every other day; if a participant had not completed the scheduled assessment the e-mail was re-sent as a prompt every day until the participant completed the assessment. If participants fell behind schedule, they were sent new links every day until they caught up, but they were instructed not to complete more than one training session per day (compliance with this instruction was also continuously monitored). Any participant who did not complete at least one training session in any 7-day period was withdrawn from the study.

After the 28 day training period, participants returned to the laboratory, logged in to their Down Your Drink account and, with the researcher’s help, used the drinking diary to populate a detailed 28-day Timeline Follow-Back drinking diary that covered the whole training period. Following this, they completed follow-up measures of the Stop Signal and Implicit Associations tasks. They were also asked “How motivated were you to reduce your alcohol consumption during the past month?” and “How would you rate your ability to reduce your alcohol consumption during the past month?” on a scale of 0–10. Participants were then partially debriefed (but group allocation was not unblinded), and reimbursed up to £130 for their participation based on attendance at the laboratory sessions and completion of training sessions (payments were not contingent on the reduction of alcohol use). All participants who completed the prespecified minimum number of training sessions (eight) and attended the three laboratory visits then self-reported their alcohol consumption using a timeline followback diary via e-mail at 2-, 4-, and 6-week follow-ups, which they were instructed to populate by accessing their drinking diaries on Down Your Drink. Participants received (up to) an additional £20 depending on the number of follow-up assessments completed. After completion of the final follow-up assessment (at 6 weeks), all participants were fully debriefed, and group allocation was unblinded. Finally, a subset of participants were randomly selected from each experimental group and invited to return to the laboratory for a short debriefing interview (see the online supplementary materials), for which they were reimbursed an additional £20.

### Data Reduction and Analysis

Our primary outcome variables were the number of U.K. units of alcohol consumed during the 4-week training period, and the number of heavy drinking days during this period. Heavy drinking days were defined as alcohol consumption >60 g (7.5 U.K. units) for males and >40 g (5 U.K. units) for females on any given day (European Medicines Agency, 2010). Our secondary outcome variable was abstinent days, defined as the number of days in which participants reported consuming no alcohol. Primary and secondary analyses were based on data from participants who attended all laboratory sessions and completed the minimum number (8) of online ICT sessions (*N* = 205).

The Down Your Drink diary was our primary source of data on participants’ alcohol consumption, but we also cross-checked this with the data that participants reported in the online platform (before each training session), and there was a robust correlation between the two (*r* = .57, *p* < .01). Note that the latter estimate is more prone to retrospective recall errors, which may account for the imperfect correlation between the two.

#### Laboratory Stop-Signal tasks

Reaction times on Go trials were subject to a trimming procedure: reaction times (RTs) faster than 200 ms or slower than 2,000 ms or those more than 3 standard deviations above the individual mean, were removed. The Stop Signal Reaction Time (SSRT) was calculated using the integration method (Verbruggen & Logan, 2009). This involves subtracting the mean SSD from the *N*th RT. The *N*th RT is calculated by multiplying the number of Go trials by the probability of inhibition errors. For example, if a participant failed to inhibit on 25% of Stop trials, the *N*th RT for this participant would be their 36th fastest Go trial (144 × 0.25 = 36). SSRT is the unobserved latency to inhibit a response, and thus larger (slower) SSRTs are indicative of poorer inhibitory control. SSRTs that were negative were removed from analyses, as these suggest that participants were waiting for stop signals rather than complying with instructions (Congdon et al., 2012).

#### Implicit Association task

IAT scores were calculated using the D600 algorithm (Greenwald, Nosek, & Banaji, 2003). Reaction times faster than 400 ms and longer than 10,000 ms were removed from analysis. Mean RTs were calculated separately for the combination blocks, including the practice blocks, after a 600 ms error penalty for incorrect responses had been applied. D600 scores were calculated as the standardized difference in RTs between blocks in such a way that a positive score indicated faster performance on blocks when alcohol pictures were paired with pleasant words compared to when alcohol pictures were paired with unpleasant words.

All data were analyzed using mixed-design ANOVAs, as detailed below. For analyses of our primary outcomes we also calculated Bayes Factors in JASP (JASP Team, 2017), using noninformed, default priors. Bayes factors of <.33 are considered evidence for the null hypothesis, >3 are considered evidence for the alternative hypothesis, and values in-between are considered as undiagnostic.

## Results

### Participant Characteristics (see Table 1) and Study Flow (Figure 1)

**Figure 1 fig1:**
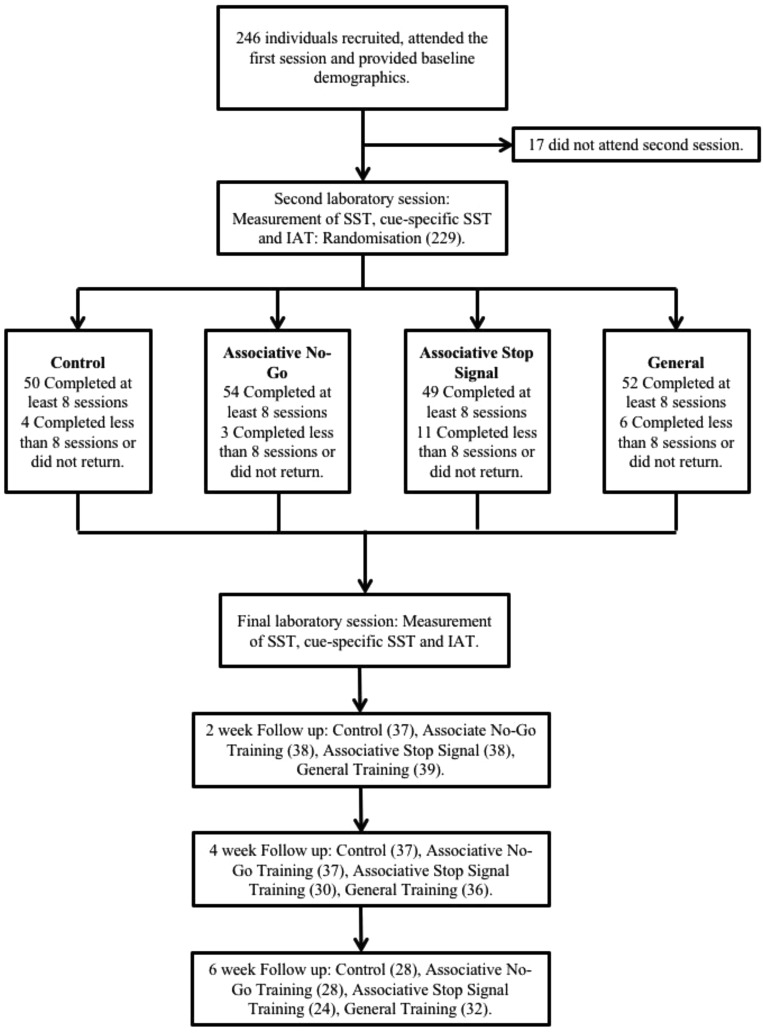
Participant flow through the study. IAT = Alcohol Valence Implicit Association Task; ICT = Inhibitory Control Training; SST = Stop Signal task.

Gender was evenly distributed across groups, χ^2^(3) = 2.17, *p* = .54. There were no differences between training completers (*N* = 205), and noncompleters (*n* = 41; comprising participants who dropped out of the study after the initial visit so were not randomized [*n* = 17], participants who were randomized but did not complete any training sessions, or did not return [*n* = 19], and participants who completed fewer than 8 ICT sessions [*n* = 5]) on any demographic variables (*t*s < 1.75, *p*s > .08). Compliance (the number of training sessions completed) was similar across groups (see the online supplementary materials).[Table-anchor tbl1][Fig-anchor fig1]

### Primary Outcomes: The Volume of Alcohol Consumed During Training (see Figure 2), and the Number of Heavy Drinking Days (Table 2)

**Figure 2 fig2:**
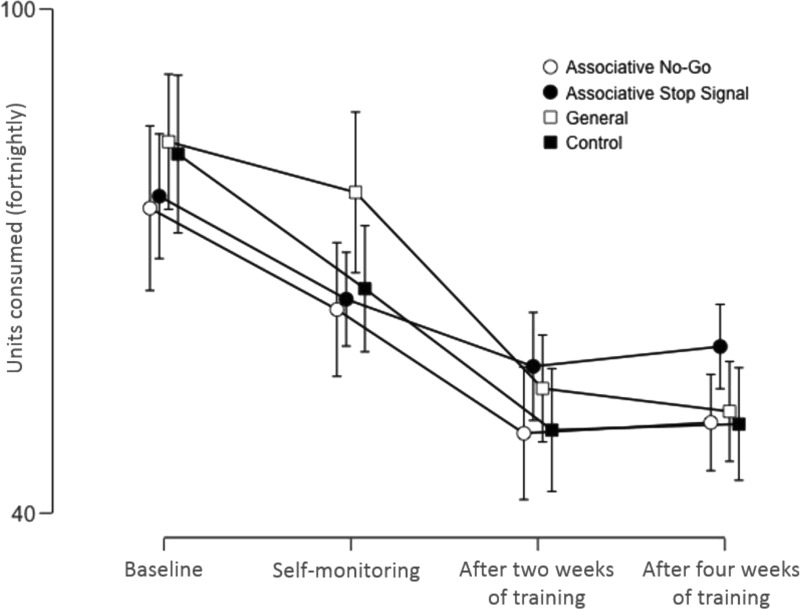
Alcohol consumption (number of units consumed per fortnight) across ICT and active control groups. Values are means (bars are 95% CIs).

The effect of training on the volume of alcohol consumed during 28 days of training was analyzed using a 4 (group: Associative No-Go, Associative Stop Signal, General, Control) × 3 (time: baseline, after 2 weeks of training, after 4 weeks of training) mixed-design ANOVA. The hypothesized time x group interaction was not significant, and Bayes factors suggest support for the null hypothesis (*F*(6, 402) = 1.12, *p* = .35, η_*p*_^2^ = .02, BF^10^ = 0.03). There was also no significant main effect of group (*F*(3, 201) = 0.45, *p* = .72, η_*p*_^2^ = .01, BF^10^ = 0.06). However, there was a significant main effect of time (*F*(2, 402) = 77.12, *p* < .01, η_*p*_^2^ = .28, BF^10^ > 99): across all groups, alcohol consumption decreased from baseline to the first 2 weeks of training, *t*(204) = 9.34, *p* < .01, *d* = 0.65, BF^10^ > 99, mean difference = 27.62 units, 95% CI [21.78. 33.45] and this reduction was maintained over the second 2 weeks (baseline vs. second 2 weeks; *t*(204) = 9.92, *p* < .01, *d* = 0.69, BF^10^ > 99, mean difference = 27.26 units, 95% CI [21.85, 32.69]), but there was no change between the first 2 weeks and second 2 weeks of training, *t*(204) = −0.20, *p* = .84, *d* = −0.01; BF^10^ = 0.07, mean difference = −0.35, 95% CI [−3.85, −3.15].[Table-anchor tbl2][Fig-anchor fig2]

The effect of training on the number of heavy drinking days was also analyzed using a 4 (group: Associative No-Go, Associative Stop Signal, General, Control) × 3 (time: baseline, after 2 weeks of training, after 4 weeks of training) mixed-design ANOVA. The hypothesized Group × Time interaction was not significant, and Bayes factors suggest support for the null hypothesis (*F*(6, 402) = 2.01, *p* = .06, η_*p*_^2^ = .03, BF^10^ = 0.22). There was no main effect of group (*F*(3, 201) = 0.63, *p* = .60, η_*p*_^2^ = .01, BF^10^ = 0.05), however there was a significant main effect of time (*F*(2, 402) = 69.48, *p* < .001, η_*p*_^2^ = .26, BF^10^ > 99): across all groups the number of heavy drinking days significantly decreased during the first 2 weeks of training (*t*(204) = 8.70, = *p* < .01, *d* = 0.60, BF^10^ > 99, mean difference = 2.16 days) and this reduction was maintained over the second 2 weeks (baseline vs. second 2 weeks; *t*(204) = 9.04, *p* < .01, *d* = 0.63, BF^10^ > 99, mean difference = 2.21 days), but there was no change between the first 2 weeks and second 2 weeks of training, *t*(204) = 0.42, *p* = .67, *d* = 0.03, BF^10^ = 0.11.

### Secondary Outcome: The Number of Abstinent Days

There were significant negative correlations between the number of abstinent days and heavy drinking days, suggesting that participants generally drank heavily on days in which they did drink (*r*s > −.50, *p*s < .01). The pattern of results for abstinent days was similar, with no significant Group × Time interaction (*F*(6, 402) = 0.20, *p* = .95, η_*p*_^2^ = .01, BF^10^ = 0.01) Full results are reported in the online supplementary materials.

### Alcohol Consumption at Follow-Up

In total, 65.86% of follow up analyses were completed: 74.27% at 2 weeks, 68.45% at 4 weeks, and 54.85% at 6 weeks. We analyzed differences in alcohol consumption at follow up using a 4 (group: Associative No-Go, Associative Stop Signal, General, Control) × 3 (time: 2-week, 4-week, 6-week follow up) ANOVA. The hypothesized Group × Time interaction was not significant (*F*(6, 154) = 0.99, *p* = .43, η_*p*_^2^ = .04). There was also no significant main effect of time (*F*(2, 154) = 0.27, *p* = .76, η_*p*_^2^ < .01). A similar pattern of results was observed for heavy drinking days and abstinent days (data not shown). Full results are reported in the online supplementary materials.

### Supplementary Analyses—The Effects of ICT on Performance on Cognitive Tasks in the Laboratory (Table 3)

#### General inhibitory control

A 4 (group: Associative No-Go, Associative Stop Signal, General, Control) × 2 (time: baseline, posttraining) mixed ANOVA tested the effects of ICT on SSRT. The hypothesized Group × Time interaction was not significant (*F*(3, 185) = 1.58, *p* = .20, η_*p*_^2^ = .03), and neither was the main effect of group (*F*(3, 185) = 1.39, *p* = .25, η_*p*_^2^ = .02). There was a significant main effect of time (*F*(1, 185) = 4.88, *p* = .03, η_*p*_^2^ = .03). SSRT was significantly faster at follow-up (202.72 ± 61.65 ms) compared to baseline (214.99 ± 58.21; *t*(189) = 2.41, *p* = .02, *d* = .18), indicative of improved inhibitory control after training in all participants.[Table-anchor tbl3]

#### Alcohol-specific inhibitory control

A 4 (group: Associative No-Go, Associative Stop Signal, General, Control) × 2 (time: baseline, posttraining) mixed ANOVA tested the effects of ICT on the disinhibiting effects of alcohol-related cues. The hypothesized Group × Time interaction was not significant (*F*(3, 190) = 1.09, *p* = .35, η_*p*_^2^ = .02). Furthermore, there were no significant main effects of time (*F*(1, 190) = 1.81, *p* = .18, η_*p*_^2^ = .01), or group (*F*(3, 190) = 0.66, *p* = .58, η_*p*_^2^ = .01).

#### Implicit Association task

One sample *t* tests comparing D600 scores to 0 ms confirmed that all participants were faster to respond on alcohol-pleasant combination blocks compared to alcohol-unpleasant combination blocks at both baseline, *t*(204) = 3.27, *p* < .01, *d* = 0.23 and posttraining, *t*(202) = 2.10, *p* = .04, *d* = 0.15, which confirms the presence of automatic associations between alcohol and positive valence in our sample. A 4 (group: Associative No-Go, Associative Stop Signal, General, Control) × 2 (time: baseline, posttraining) mixed ANOVA examined the effects of ICT on these D600 scores. The hypothesized Time × Group interaction was not significant (*F*(3, 199) = 2.08, *p* = .11, η_*p*_^2^ = .03). Furthermore, there were no significant main effects of time (*F*(1, 199) = 1.31, *p* = .26, η_*p*_^2^ = .01) or group (*F*(3, 199) = 0.14, *p* = .93, η_*p*_^2^ < .01).

## Discussion

This was the first preregistered RCT to contrast different types of ICT for their sustained effects on alcohol consumption in heavy drinkers who were motivated to reduce their drinking. We observed moderate reductions in our primary alcohol consumption outcome measures (total consumption and the number of heavy drinking days); however, the absence of any differential effectiveness of ICT versus active control suggests no beneficial effects that can be directly attributed to ICT. Furthermore, although we observed a small improvement in inhibitory control capacity after training in all participants, there were no differential effects of ICT versus active control on this or any of the other candidate psychological mechanisms of action of ICT, including changes in devaluation of alcohol cues, or improvements in cue-specific inhibitory control.

Our primary hypothesis that all forms of ICT would reduce alcohol consumption compared to a control intervention was not supported, and Bayes factors were strongly in favor of the null hypothesis. These findings demonstrate the complexity of translation of promising and consistent findings from laboratory studies (Allom et al., 2016; Jones et al., 2016) into a novel behavior change intervention that can help heavy drinkers to reduce their alcohol consumption in the real world. Furthermore, our findings are surprising given that applications of associative ICT in other domains, such as food-related ICT to aid weight loss, have been successfully translated from the laboratory to real-world settings (Allom & Mullan, 2015; Lawrence et al., 2015; Veling et al., 2014; but see Allom & Mullan, 2015, Study 2, for a failure to replicate). Our results were also not impacted by stratification of participants into those who reduced their alcohol intake after a brief intervention and a week of self-monitoring (“early reducers”), and those who did not. This is in contrast with previous research, which demonstrated that a floor effect among early reducers may have masked the effects of nalmefene on drinking outcomes (Gual et al., 2013). However, our sample comprised a larger proportion of early reducers than Gual et al., which may be attributable to characteristics of the different samples.

We demonstrated an overall reduction in alcohol consumption across all experimental groups, which is typical in designs where intervention groups are contrasted against active control groups. This reduction is likely to be attributable to features of the intervention that were common to all participants (including the active control group), including exposure to a brief intervention before randomization, regular self-monitoring of alcohol consumption (Jenkins, McAlaney, & McCambridge, 2009; McCambridge, Kypri, & McElduff, 2014; McCambridge & Kypri, 2011), and repeated engagement with an ostensibly active intervention, with financial incentives that were proportionate to engagement with the intervention (Giles, Robalino, McColl, Sniehotta, & Adams, 2014). Indeed, we observed that, across the whole sample, participants who completed more training sessions tended to report larger reductions in drinking over the course of the training period (see the online supplementary materials). This might indicate that participants who were most motivated to engage with the intervention were most likely to reduce their drinking in response to it. Although interesting in its own right (Jenkins et al., 2009), this pronounced nonspecific reduction in alcohol consumption over the course of the training period (∼ 27 units per fortnight, from baseline to the final 2 weeks of training: *d* = 0.69) may have obscured any additional beneficial effects of ICT, which has demonstrably smaller effect sizes in other domains (e.g., pre–post training comparison of weight change in Lawrence et al., (2015) was *d = .40*). Indeed, a recent evaluation of ICT for sleep hygiene behaviors noted that the beneficial effects of (general) ICT may have been obscured by the pronounced beneficial effects of self-monitoring (Todd & Mullan, 2014). One implication is that outside of the laboratory setting, ICT has no incremental effect on alcohol consumption beyond these potent nonspecific effects. These nonspecific effects of the intervention that were common to all participants (including the active control group), specifically completion of the Down Your Drink intervention followed by the period of regular self-monitoring of alcohol consumption, may have effectively served as an alternative form of inhibitory training that could have obscured any additional benefits of the computerized ICT that we evaluated in this study. This possibility could be investigated in future research by comparing ICT with a different control condition, preferably one that does not involve self-monitoring of behavior. Although such a trial may provide a more “pure” empirical test of the effects of computerized ICT, the intervention would be unlikely to actually be implemented in this way (i.e., in isolation), given that self-monitoring and motivational enhancement are much easier to administer and much less demanding for participants, compared to computerized ICT. This highlights a practical issue regarding translation of laboratory findings into viable behavior change interventions: to what extent should evaluations of those interventions be divorced from the broader context in which those interventions are ultimately likely to be delivered? Similarly, participants had no face-to-face contact with the researchers during the ICT training period (although there was personal contact immediately before and immediately after the training period). This may also partially account for the observed null effects of ICT, because computer-delivered interventions tend to yield larger effects if there is some face-to-face contact during the intervention period (Black, Mullan, & Sharpe, 2016). This issue could be investigated in future research. However, we note that if ICT requires face-to-face contact with a clinician or researcher in order to be effective, this would drastically limit the feasibility of its widespread implementation and its cost effectiveness.

Aside from a nonspecific improvement in inhibitory control in all participants (which may reflect a practice effect rather than an effect of ICT per se), none of our candidate psychological mechanisms of action were influenced by the different variants of ICT. Specifically, neither Associative No-Go nor Stop Signal training prompted changes in the disinhibiting effects of alcohol cues or in automatic affective responses to those cues. However, although we opted to use the IAT to measure devaluation effects on the basis of published studies at the time that we planned the study (Houben et al., 2011, 2012), work published in the interim suggests that the effects of ICT on this task are not robust (see Jones et al., 2016), and devaluation effects after ICT may only be detectable with self-report measures (Chen et al., 2016; Lawrence et al., 2015; Veling et al., 2013). Regardless, in the context of the broader literature on Cognitive Bias Modification (CBM), a class of behavioral interventions to which ICT belongs, the failure of ICT to influence the psychological constructs that it targeted makes the null effects on alcohol consumption difficult to interpret (see MacLeod & Grafton, 2016; Wiers, Boffo, & Field, in press), an issue that we revisit below.

Our findings should be interpreted in the context of possible limitations of our methodology. First, it is possible that our Stop Signal training paradigms were simply inadequate to produce sustained improvements in inhibitory control capacity or the disinhibiting effects of alcohol cues. Most participants in these groups successfully inhibited responding on the majority of Stop Signal trials, even though the task got progressively more difficult as training progressed. This suggests that the tasks may have been too easy. Future studies could investigate alternative methods for adjusting the difficulty of Stop Signal training in order to train inhibitory control over multiple sessions (e.g., Berkman, Kahn & Merchant (2014)). However, findings from a recent meta-analysis (Jones et al., 2016) suggest that the magnitude of the effect of ICT on consummatory behavior is closely related to the proportion of successful inhibitions to appetitive cues. Therefore, successful inhibition of behavior during exposure to target cues may be more important than maximizing the difficulty of inhibition (which would inevitably result in a higher proportion of inhibitory failures). Second, the contingency between alcohol pictures and stop signals in Associative Stop Signal training (50%) may have been suboptimal for the establishment of alcohol-inhibition associations (see Jones et al., 2016; Verbruggen, Best, Bowditch, Stevens, & McLaren, 2014). However, this contingency was 100% in the Associative No-Go training group, which renders this an unlikely explanation for the null effects on drinking outcomes. Third, self-reported alcohol consumption may underestimate actual consumption (Boniface, Kneale, & Shelton, 2014), and it is sensitive to social desirability and recall errors (Gmel & Daeppen, 2007). To mitigate these concerns, participants were instructed to record their alcohol consumption online at the end of every day during the training period, and these data showed good convergent validity with alcohol consumption that participants self-reported at the beginning of each training session (every other day, on average). Furthermore, biases associated with social desirability, underestimation, and recall errors should have affected all participants equally (regardless of group allocation). Nonetheless, future studies of this type should corroborate self-reported alcohol consumption using objective measures, such as transdermal alcohol sensors (Neville, Williams, Goodall, Murer, & Donnelly, 2013).

Our study also has strengths. First, we preregistered our methodology, hypotheses, and analysis strategy, which should increase confidence in the transparency of our analytic strategy and the likely replicability of our findings (Munafò et al., 2017). Second, it was more than adequately powered to test our primary hypotheses (unlike many lab-based studies; see Jones et al., 2018). We also used an “active” control group, which is often lacking and is a pervasive problem in evaluations of psychological interventions (Boot, Simons, Stothart, & Stutts, 2013). Indeed, the absence of an active control seems likely to have artificially inflated the apparent effectiveness of similar interventions for this population (Cox, Fadardi, Hosier, & Pothos, 2015; Fadardi & Cox, 2009). Finally, we contrasted different variants of ICT with each other, and comprehensively investigated changes in candidate psychological mechanisms of action (Stice et al., 2016), issues which are often overlooked in standard efficacy trials.

Future research on ICT (and other forms of cognitive bias modification for alcohol problems) could benefit by refining treatment protocols before conducting RCTs, which are time consuming and expensive. For example, in the case of Associative ICT it is important to ensure that participants learn to form direct stimulus–response associations between alcohol-related cues and inhibition of behavior, but existing ICT paradigms may teach participants to associate alcohol-related cues with signals to inhibit rather than inhibition of behavior itself. This distinction is important because the latter learning is unlikely to generalize across different contexts, and therefore unlikely to generalize to actual drinking behavior (Boutelle & Bouton, 2015; Verbruggen, McLaren, & Chambers, 2014). One way to achieve this might be to use multiple different inhibition signals in order to strengthen direct stimulus–response associations (see Best, Lawrence, Logan, McLaren, & Verbruggen, 2016). Future research could also make ICT more engaging by gamifying it and/or presenting it on smartphone apps (see Blackburne et al., 2016; Boendermaker, Prins, & Wiers, 2015), as highlighted by participants during debriefing after the trial (see the online supplementary materials). However, this is not straightforward, because if gamification is poorly implemented it can reduce motivation and compliance (Boendermaker, Sanchez Maceiras, Boffo, & Wiers, 2016). It may also be important to pair inhibition with images of participants’ preferred beverages, rather than images of a range of different alcoholic drinks (cf. Fadardi & Cox, 2009).

Finally, our primary and secondary outcome measures were obtained from participants’ self-reports of their alcohol consumption, as is common in the alcohol field (including in other trials of CBM interventions, including ICT, e.g., Eberl et al., 2013; Fadardi & Cox, 2009; Houben et al., 2011, 2012; Wiers, Eberl, Rinck, Becker, & Lindenmeyer, 2011). Methodological concerns about reliance on self-report, including participant demand effects and reliance on recall over prolonged periods of time (Midanik, 1982), should be minimized given that participants completed drinking diaries at the end of each day or the beginning of the next, which maximizes the accuracy of self-report estimates (Carney, Tennen, Affleck, Del Boca, & Kranzler, 1998; Ekholm, 2004). Participants were trained on how to use the daily alcohol diary before the training period, which may have contributed to the excellent compliance (Shiffman, 2009). We also note that self-reported alcohol consumption has good agreement with estimates from observers (“collaterals”; Babor, Steinberg, Anton, & Del Boca, 2000). Future studies might attempt to corroborate findings with objective measures of alcohol intake, such as bogus taste tests (Jones, Button et al., 2016), as these are known to be sensitive to the effects of a single session of ICT (Allom et al., 2016; Jones, Di Lemma et al., 2016). However, these laboratory measurements are primarily intended to provide behavioral indices of alcohol consumption in “proof of concept” laboratory studies, and indeed one may question the ethics of providing alcohol to participants who are recruited to an intervention to reduce their alcohol consumption.

To conclude, we found that Internet-delivered ICT does not help problem drinkers to reduce their alcohol consumption beyond the nonspecific effects associated with taking part in a trial. These null findings are in line with those from recent meta-analyses that suggest negligible clinical utility of CBM interventions for addiction (Cristea, Kok, & Cuijpers, 2016) and other psychological disorders (Cristea, Kok, & Cuijpers, 2015), although this pessimistic conclusion may overlook important details regarding when CBM is likely to be effective and when it is not (MacLeod & Grafton, 2016; Wiers et al., in press). Future laboratory research should attempt to refine ICT in the laboratory in order to ensure that it effectively engages its target psychological constructs. However, it is important to remain open to the possibility that ICT may not translate into an effective behavior change intervention, even with extensive modifications to training procedures.

## Supplementary Material

10.1037/ccp0000312.supp

## Figures and Tables

**Table 1 tbl1:** Demographic and Questionnaire Variables at Baseline

Experimental group	Control	Associative No-Go	Associative stop	General	Nonrandomized
Gender (M/F)	25/29	33/24	32/28	32/26	8/9
Age	42.42 (12.35)	39.43 (10.67)	43.88 (11.89)	40.64 (11.93)	37.41 (10.79)
Alcohol consumption (units)	81.55 (50.94)	77.81 (43.22)	78.93 (39.69)	87.88 (51.31)	67.93 (38.66)
AUDIT	15.85 (6.68)	14.42 (5.98)	15.00 (6.27)	13.91 (5.16)	15.25 (6.12)
BIS–attention	19.15 (3.93)	17.54 (3.32)	18.52 (3.35)	18.16 (2.96)	18.82 (4.16)
BIS–nonplanning	26.70 (5.16)	24.79 (4.50)	25.57 (5.24)	25.74 (4.82)	27.65 (5.82)
BIS–motor	24.05 (4.39)	22.93 (3.72)	23.72 (3.69)	23.78 (4.10)	23.71 (4.12)
TRI–CEP	35.24 (16.97)	29.67 (13.76)	35.78 (14.30)	32.45 (13.88)	30.76 (14.35)
TRI–CBC	22.42 (9.92)	22.35 (10.11)	23.75 (8.97)	22.50 (9.29)	22.24 (10.46)
*Note*. Values are means (standard deviations). AUDIT = Alcohol Use Disorders Identification Test; BIS = Barratt Impulsiveness Scale; TRI = Temptation and Restraint Inventory; Alcohol consumption = alcohol consumption in UK units (1 UK Unit = 8 g of pure alcohol), per fortnight. The Nonrandomized group refers to participants who attended the first session, but did not return to the laboratory after the initial week of self-monitoring.					

**Table 2 tbl2:** Mean Number of Heavy Drinking Days Per Fortnight Across Time Points and Groups

Experimental group	Control	Assoc. No-Go	Assoc. stop	General
Baseline	5.38 (3.33)	5.41 (3.89)	5.16 (3.57)	6.19 (3.61)
First 2 weeks training	3.26 (2.51)	2.96 (2.59)	3.84 (3.31)	3.50 (2.88)
Second 2weeks training	3.04 (2.06)	3.06 (2.67)	4.10 (3.04)	3.15 (2.85)
*Note*. Values are means (standard deviations).				

**Table 3 tbl3:** Inhibitory Control and Alcohol Affective Associations Over Time and Across Groups

Experimental group	Control	Assoc. No-Go	Assoc. stop	General
General SSRT (pre)	225.50 (62.85)	207.51 (49.71)	227.60 (61.34)	204.96 (57.50)
General SSRT (post)	202.05 (40.04)	212.30 (67.53)	203.93 (61.66)	190.05 (67.21)
Alcohol SSRT (pre)	217.19 (46.49)	224.30 (72.74)	210.01 (64.30)	200.47 (47.08)
Alcohol SSRT (post)	204.84 (58.88)	208.87 (62.54)	198.32 (56.08)	209.01 (85.41)
D600 (pre)	.11 (.63)	.14 (.42)	.14 (.39)	.06 (.49)
D600 (post)	.04 (.65)	.02 (.48)	.08 (.44)	.17 (.50)
*Note*. Values are means (standard deviations). SSRT = Stop Signal Reaction Time.				

## Footnotes

1 The labels for the different forms of ICT differ from those used in the published study protocol. This reflects developments in our understanding of ICT since the protocol was published (Jones et al., 2014), and we hope that these labels will clarify the similarities and differences between the different forms of ICT.

2 Our preregistered effect size of interest (*f* = .25) was conducted before subsequent studies were published, which demonstrated larger effects of repeated sessions of ICT on behavior outside of the laboratory (*f* ∼ .29; Lawrence et al. (2015)), which suggests that our power calculation was conservative.

3 Note, this research was initiated and completed before the United Kingdom guidelines were revised to 14 units per week for both males and females in January 2016.

## References

[c1] Allom V., & Mullan B. (2015). Two inhibitory control training interventions designed to improve eating behaviour and determine mechanisms of change. Appetite, 89, 282–290. 10.1016/j.appet.2015.02.022 25725487

[c2] Allom V., Mullan B., & Hagger M. (2016). Does inhibitory control training improve health behaviour? A meta-analysis. Health Psychology Review, 10, 168–186. 10.1080/17437199.2015.1051078 26058688

[c3] Babor T. F., Higgins-Biddle J. C., Saunders J. B., & Monteiro M. G. (2001). The Alcohol Use Disorders Identification Test: Guidelines for use in primary care. Geneva, Switzerland: World Health Organization.

[c4] Babor T. F., Steinberg K., Anton R., & Del Boca F. (2000). Talk is cheap: Measuring drinking outcomes in clinical trials. Journal of Studies on Alcohol, 61, 55–63. 10.15288/jsa.2000.61.55 10627097

[c5] Barry K. L., Blow F. C., Willenbring M. L., McCormick R., Brockmann L. M., & Visnic S. (2004). Use of alcohol screening and brief interventions in primary care settings: Implementation and barriers. Substance Abuse, 25, 27–36. 10.1300/J465v25n01_05 15201109

[c6] Bartsch A. L., Kothe E., Allom V., Mullan B., & Houben K. (2016). The effect of non-specific response inhibition training on alcohol consumption: An intervention. Journal of Addiction Research & Therapy, 7, 260 10.4172/2155-6105.1000260

[c94] Berkman E. T., Kahn L. E., & Merchant J. S. (2014). Training-induced changes in inhibitory control network activity. The Journal of Neuroscience, 34, 149–157. 24381276 10.1523/JNEUROSCI.3564-13.2014 PMC3866481

[c7] Best M., Lawrence N. S., Logan G. D., McLaren I. P. L., & Verbruggen F. (2016). Should I stop or should I go? The role of associations and expectancies. Journal of Experimental Psychology: Human Perception and Performance, 42, 115–137. 10.1037/xhp0000116 26322688 PMC4685931

[c8] Bickel W. K., Jarmolowicz D. P., Mueller E. T., Gatchalian K. M., & McClure S. M. (2012). Are executive function and impulsivity antipodes? A conceptual reconstruction with special reference to addiction. Psychopharmacology, 221, 361–387. 10.1007/s00213-012-2689-x 22441659 PMC4035182

[c9] Black N., Mullan B., & Sharpe L. (2016). Computer-delivered interventions for reducing alcohol consumption: Meta-analysis and meta-regression using behaviour change techniques and theory. Health Psychology Review, 10, 341–357. 10.1080/17437199.2016.1168268 26999311

[c10] Blackburne T., Rodriguez A., & Johnstone S. J. (2016). A serious game to increase healthy food consumption in overweight or obese adults: Randomized controlled trial. JMIR Serious Games, 4, e10 10.2196/games.5708 27417192 PMC4963607

[c11] Boendermaker W. J., Prins P. J., & Wiers R. W. (2015). Cognitive Bias Modification for adolescents with substance use problems—Can serious games help? Journal of Behavior Therapy and Experimental Psychiatry, 49, 13–20. 10.1016/j.jbtep.2015.03.008 25843611

[c12] Boendermaker W. J., Sanchez Maceiras S., Boffo M., & Wiers R. W. (2016). Attentional Bias Modification with serious game elements: Evaluating the Shots game. JMIR Serious Games, 4, e20 10.2196/games.6464 27923780 PMC5174726

[c13] Boniface S., Kneale J., & Shelton N. (2014). Drinking pattern is more strongly associated with under-reporting of alcohol consumption than socio-demographic factors: Evidence from a mixed-methods study. BMC Public Health, 14, 1297 10.1186/1471-2458-14-1297 25519144 PMC4320509

[c14] Boot W. R., Simons D. J., Stothart C., & Stutts C. (2013). The pervasive problem with placebos in psychology: Why active control groups are not sufficient to rule out placebo effects. Perspectives on Psychological Science, 8, 445–454. 10.1177/1745691613491271 26173122

[c15] Boutelle K. N., & Bouton M. E. (2015). Implications of learning theory for developing programs to decrease overeating. Appetite, 93, 62–74. 10.1016/j.appet.2015.05.013 25998235 PMC4654402

[c16] Carney M. A., Tennen H., Affleck G., Del Boca F. K., & Kranzler H. R. (1998). Levels and patterns of alcohol consumption using timeline follow-back, daily diaries and real-time “electronic interviews”. Journal of Studies on Alcohol, 59, 447–454. 10.15288/jsa.1998.59.447 9647427

[c17] Carter B. L., & Tiffany S. T. (1999). Meta-analysis of cue-reactivity in addiction research. Addiction, 94, 327–340. 10.1046/j.1360-0443.1999.9433273.x 10605857

[c18] Chen Z., Veling H., Dijksterhuis A., & Holland R. W. (2016). How does not responding to appetitive stimuli cause devaluation: Evaluative conditioning or response inhibition? Journal of Experimental Psychology: General, 145, 1687–1701. 10.1037/xge0000236 27736134

[c19] Christiansen P., Cole J. C., Goudie A. J., & Field M. (2012). Components of behavioural impulsivity and automatic cue approach predict unique variance in hazardous drinking. Psychopharmacology, 219, 501–510. 10.1007/s00213-011-2396-z 21735071

[c20] Collins R. L., & Lapp W. M. (1992). The Temptation and Restraint Inventory for measuring drinking restraint. British Journal of Addiction, 87, 625–633. 10.1111/j.1360-0443.1992.tb01964.x 1591514

[c21] Congdon E., Mumford J. A., Cohen J. R., Galvan A., Canli T., & Poldrack R. A. (2012). Measurement and reliability of response inhibition. Frontiers in Psychology, 3, 37 10.3389/fpsyg.2012.00037 22363308 PMC3283117

[c22] Cox W. M., Fadardi J. S., Hosier S. G., & Pothos E. M. (2015). Differential effects and temporal course of attentional and motivational training on excessive drinking. Experimental and Clinical Psychopharmacology, 23, 445–454. 10.1037/pha0000038 26348159 PMC4655870

[c23] Cristea I. A., Kok R. N., & Cuijpers P. (2015). Efficacy of cognitive bias modification interventions in anxiety and depression: Meta-analysis. The British Journal of Psychiatry, 206, 7–16. 10.1192/bjp.bp.114.146761 25561486

[c24] Cristea I. A., Kok R. N., & Cuijpers P. (2016). The effectiveness of cognitive bias modification interventions for substance addictions: A meta-analysis. PLoS ONE, 11(9), e0162226 10.1371/journal.pone.0162226 27611692 PMC5017662

[c25] Czapla M., Simon J. J., Richter B., Kluge M., Friederich H. C., Herpertz S., . . . Loeber S. (2016). The impact of cognitive impairment and impulsivity on relapse of alcohol-dependent patients: Implications for psychotherapeutic treatment. Addiction Biology, 21, 873–884. 10.1111/adb.12229 25678237

[c95] Di Lemma L. C. G., & Field M. (2017). Cue avoidance training and inhibitory control training for the reduction of alcohol consumption: A comparison of effectiveness and investigation of their mechanisms of action. Psychopharmacology, 234, 2489–2498. 28551714 10.1007/s00213-017-4639-0 PMC5537323

[c26] de Wit H. (2009). Impulsivity as a determinant and consequence of drug use: A review of underlying processes. Addiction Biology, 14, 22–31. 10.1111/j.1369-1600.2008.00129.x 18855805 PMC3640851

[c27] Eagle D. M., Bari A., & Robbins T. W. (2008). The neuropsychopharmacology of action inhibition: Cross-species translation of the stop-signal and go/no-go tasks. Psychopharmacology, 199, 439–456. 10.1007/s00213-008-1127-6 18542931

[c28] Eberl C., Wiers R. W., Pawelczack S., Rinck M., Becker E. S., & Lindenmeyer J. (2013). Approach bias modification in alcohol dependence: Do clinical effects replicate and for whom does it work best? Developmental Cognitive Neuroscience, 4, 38–51. 10.1016/j.dcn.2012.11.002 23218805 PMC6987692

[c29] Edwards G. (1996). Sensible drinking: Doctors should stick with the independent medical advice. British Medical Journal, 312, 1 10.1136/bmj.312.7022.1 8555842 PMC2349701

[c96] Ekholm O. (2004). Influence of the recall period on self-reported alcohol intake. European Journal of Clinical Nutrition, 58, 60–63. 14679368 10.1038/sj.ejcn.1601746

[c30] European Medicines Agency (2010). Guidelines on the development of medicinal products for the treatment of alcohol dependence. London, UK: Author.

[c31] Fadardi J. S., & Cox W. M. (2009). Reversing the sequence: Reducing alcohol consumption by overcoming alcohol attentional bias. Drug and Alcohol Dependence, 101, 137–145. 10.1016/j.drugalcdep.2008.11.015 19193499

[c32] Field M., & Jones A. (2017). Elevated alcohol consumption following alcohol cue exposure is partially mediated by reduced inhibitory control and increased craving. Psychopharmacology, 234, 2979–2988. 10.1007/s00213-017-4694-6 28741032 PMC5591800

[c33] Friese M., Frankenbach J., Job V., & Loschelder D. D. (2017). Does self-control training improve self-control? A meta-analysis. Perspectives on Psychological Science, 12, 1077–1099. 10.1177/1745691617697076 28846503

[c34] Fujita K. (2011). On conceptualizing self-control as more than the effortful inhibition of impulses. Personality and Social Psychology Review, 15, 352–366. 10.1177/1088868311411165 21685152

[c35] Gauggel S., Heusinger A., Forkmann T., Boecker M., Lindenmeyer J., Cox W. M., & Staedtgen M. (2010). Effects of alcohol cue exposure on response inhibition in detoxified alcohol-dependent patients. Alcoholism: Clinical and Experimental Research, 34, 1584–1589. 10.1111/j.1530-0277.2010.01243.x 20586755

[c36] Giles E. L., Robalino S., McColl E., Sniehotta F. F., & Adams J. (2014). The effectiveness of financial incentives for health behaviour change: Systematic review and meta-analysis. PLoS ONE, 9(3), e90347 10.1371/journal.pone.0090347 24618584 PMC3949711

[c37] Gmel G., & Daeppen J. B. (2007). Recall bias for seven-day recall measurement of alcohol consumption among emergency department patients: Implications for case-crossover designs. Journal of Studies on Alcohol and Drugs, 68, 303–310. 10.15288/jsad.2007.68.303 17286350

[c38] Goldstein R. Z., & Volkow N. D. (2011). Dysfunction of the prefrontal cortex in addiction: Neuroimaging findings and clinical implications. Nature Reviews Neuroscience, 12, 652–669. 10.1038/nrn3119 22011681 PMC3462342

[c39] Greenwald A. G., Nosek B. A., & Banaji M. R. (2003). Understanding and using the implicit association test: I. An improved scoring algorithm. Journal of Personality and Social Psychology, 85, 197–216. 10.1037/0022-3514.85.2.197 12916565

[c40] Gual A., He Y., Torup L., van den Brink W., & Mann K. (2013). A randomised, double-blind, placebo-controlled, efficacy study of nalmefene, as-needed use, in patients with alcohol dependence. European Neuropsychopharmacology, 23, 1432–1442. 10.1016/j.euroneuro.2013.02.006 23562264

[c41] Hofmann W., Friese M., & Strack F. (2009). Impulse and self-control from a dual-systems perspective. Perspectives on Psychological Science, 4, 162–176. 10.1111/j.1745-6924.2009.01116.x 26158943

[c42] Hofmann W., Schmeichel B. J., & Baddeley A. D. (2012). Executive functions and self-regulation. Trends in Cognitive Sciences, 16, 174–180. 10.1016/j.tics.2012.01.006 22336729

[c43] Houben K., Havermans R. C., Nederkoorn C., & Jansen A. (2012). Beer à no-go: Learning to stop responding to alcohol cues reduces alcohol intake via reduced affective associations rather than increased response inhibition. Addiction, 107, 1280–1287. 10.1111/j.1360-0443.2012.03827.x 22296168

[c44] Houben K., Nederkoorn C., Wiers R. W., & Jansen A. (2011). Resisting temptation: Decreasing alcohol-related affect and drinking behavior by training response inhibition. Drug and Alcohol Dependence, 116, 132–136. 10.1016/j.drugalcdep.2010.12.011 21288663

[c45] Houston R. J., Derrick J. L., Leonard K. E., Testa M., Quigley B. M., & Kubiak A. (2014). Effects of heavy drinking on executive cognitive functioning in a community sample. Addictive Behaviors, 39, 345–349. 10.1016/j.addbeh.2013.09.032 24459697 PMC4101901

[c97] JASP Team (2017). JASP (Version 0.8.4.0) [Computer software]. Retrieved from https://jasp-stats.org/

[c46] Jenkins R. J., McAlaney J., & McCambridge J. (2009). Change over time in alcohol consumption in control groups in brief intervention studies: Systematic review and meta-regression study. Drug and Alcohol Dependence, 100, 107–114. 10.1016/j.drugalcdep.2008.09.016 19041196

[c47] Jin J., Sklar G. E., Oh V. M. S., & Li S. C. (2008). Factors affecting therapeutic compliance: A review from the patient’s perspective. Therapeutics and Clinical Risk Management, 4, 269–286. 10.2147/TCRM.S1458 18728716 PMC2503662

[c48] Jones A., Button E., Rose A. K., Robinson E., Christiansen P., Di Lemma L., & Field M. (2016). The ad-libitum alcohol ‘taste test’: Secondary analyses of potential confounds and construct validity. Psychopharmacology, 233, 917–924. 10.1007/s00213-015-4171-z 26680342 PMC4751185

[c49] Jones A., Cole J., Goudie A., & Field M. (2011). Priming a restrained mental set reduces alcohol-seeking independently of mood. Psychopharmacology, 218, 557–565. 10.1007/s00213-011-2338-9 21603894

[c50] Jones A., Di Lemma L. C. G., Robinson E., Christiansen P., Nolan S., Tudur-Smith C., & Field M. (2016). Inhibitory control training for appetitive behaviour change: A meta-analytic investigation of mechanisms of action and moderators of effectiveness. Appetite, 97, 16–28. 10.1016/j.appet.2015.11.013 26592707

[c51] Jones A., & Field M. (2013). The effects of cue-specific inhibition training on alcohol consumption in heavy social drinkers. Experimental and Clinical Psychopharmacology, 21, 8–16. 10.1037/a0030683 23181512

[c52] Jones A., & Field M. (2015). Alcohol-related and negatively valenced cues increase motor and oculomotor disinhibition in social drinkers. Experimental and Clinical Psychopharmacology, 23, 122–129. 10.1037/pha0000011 25730418 PMC4386809

[c53] Jones A., Guerrieri R., Fernie G., Cole J., Goudie A., & Field M. (2011). The effects of priming restrained versus disinhibited behaviour on alcohol-seeking in social drinkers. Drug and Alcohol Dependence, 113, 55–61. 10.1016/j.drugalcdep.2010.07.006 20724083

[c54] Jones A., Hardman C. A., Lawrence N., & Field M. (2018). Cognitive training as a potential treatment for overweight and obesity: A critical review of the evidence. Appetite, 124, 50–67. 10.1016/j.appet.2017.05.032 28546010

[c55] Jones A., McGrath E., Houben K., Nederkoorn C., Robinson E., & Field M. (2014). A comparison of three types of web-based inhibition training for the reduction of alcohol consumption in problem drinkers: Study protocol. BMC Public Health, 14, 796 10.1186/1471-2458-14-796 25090915 PMC4131042

[c56] Kaner E., Bland M., Cassidy P., Coulton S., Dale V., Deluca P., . . . Drummond C. (2013). Effectiveness of screening and brief alcohol intervention in primary care (SIPS trial): Pragmatic cluster randomised controlled trial. British Medical Journal, 346, e8501 10.1136/bmj.e8501 23303891 PMC3541471

[c57] Lawrence A. J., Luty J., Bogdan N. A., Sahakian B. J., & Clark L. (2009). Impulsivity and response inhibition in alcohol dependence and problem gambling. Psychopharmacology, 207, 163–172. 10.1007/s00213-009-1645-x 19727677 PMC2764851

[c58] Lawrence N. S., O’Sullivan J., Parslow D., Javaid M., Adams R. C., Chambers C. D., . . . Verbruggen F. (2015). Training response inhibition to food is associated with weight loss and reduced energy intake. Appetite, 95, 17–28. 10.1016/j.appet.2015.06.009 26122756 PMC4596151

[c59] Linke S., Brown A., & Wallace P. (2004). Down your drink: A web-based intervention for people with excessive alcohol consumption. Alcohol and Alcoholism, 39, 29–32. 10.1093/alcalc/agh004 14691071

[c60] Logan G. D., Cowan W. B., & Davis K. A. (1984). On the ability to inhibit simple and choice reaction time responses: A model and a method. Journal of Experimental Psychology: Human Perception and Performance, 10, 276–291. 10.1037/0096-1523.10.2.276 6232345

[c61] MacLeod C., & Grafton B. (2016). Anxiety-linked attentional bias and its modification: Illustrating the importance of distinguishing processes and procedures in experimental psychopathology research. Behaviour Research and Therapy, 86, 68–86. 10.1016/j.brat.2016.07.005 27461003

[c62] McCambridge J., & Kypri K. (2011). Can simply answering research questions change behaviour? Systematic review and meta-analyses of brief alcohol intervention trials. PLoS ONE, 6(10), e23748 10.1371/journal.pone.0023748 21998626 PMC3187747

[c63] McCambridge J., Kypri K., & McElduff P. (2014). Regression to the mean and alcohol consumption: A cohort study exploring implications for the interpretation of change in control groups in brief intervention trials. Drug and Alcohol Dependence, 135, 156–159. 10.1016/j.drugalcdep.2013.11.017 24342421 PMC3929002

[c64] Midanik L. (1982). The validity of self-reported alcohol consumption and alcohol problems: A literature review. British Journal of Addiction, 77, 357–382. 10.1111/j.1360-0443.1982.tb02469.x 6762224

[c65] Miyake A., Friedman N. P., Emerson M. J., Witzki A. H., Howerter A., & Wager T. D. (2000). The unity and diversity of executive functions and their contributions to complex “Frontal Lobe” tasks: A latent variable analysis. Cognitive Psychology, 41, 49–100. 10.1006/cogp.1999.0734 10945922

[c66] Munafò M., Nosek B. A., Bishop D., Button K. S., Chambers C. D., Percie du Sert N., . . . Ioannidis J. P. A. (2017). A manifesto for reproducible science. Nature Human Behaviour, 1, 0021 10.1038/s41562-016-0021 PMC7610724 33954258

[c67] Neville F. G., Williams D. J., Goodall C. A., Murer J. S., & Donnelly P. D. (2013). An experimental trial exploring the impact of continuous transdermal alcohol monitoring upon alcohol consumption in a cohort of male students. PLoS ONE, 8(6), e67386 10.1371/journal.pone.0067386 23825656 PMC3692417

[c68] Patton J. H., Stanford M. S., & Barratt E. S. (1995). Factor structure of the Barratt impulsiveness scale. Journal of Clinical Psychology, 51, 768–774. 10.1002/1097-4679(199511)51:6<768::AID-JCLP2270510607>3.0.CO;2-1 8778124

[c69] Petit G., Kornreich C., Noël X., Verbanck P., & Campanella S. (2012). Alcohol-related context modulates performance of social drinkers in a visual Go/No-Go task: A preliminary assessment of event-related potentials. PLoS ONE, 7(5), e37466 10.1371/journal.pone.0037466 22616012 PMC3355129

[c70] Rehm J., Mathers C., Popova S., Thavorncharoensap M., Teerawattananon Y., & Patra J. (2009). Global burden of disease and injury and economic cost attributable to alcohol use and alcohol-use disorders. The Lancet, 373, 2223–2233. 10.1016/S0140-6736(09)60746-7 19560604

[c71] Riper H., Spek V., Boon B., Conijn B., Kramer J., Martin-Abello K., & Smit F. (2011). Effectiveness of E-self-help interventions for curbing adult problem drinking: A meta-analysis. Journal of Medical Internet Research, 13, e42 10.2196/jmir.1691 21719411 PMC3221381

[c72] Shiffman S. (2009). Ecological momentary assessment (EMA) in studies of substance use. Psychological Assessment, 21, 486–497. 10.1037/a0017074 19947783 PMC2846437

[c98] Smith J. L., Dash N. J., Johnstone S. J., Houben K., & Field M. (2017). Current forms of inhibitory training produce no greater reduction in drinking than simple assessment: A preliminary study. Drug and Alcohol Dependence, 173, 47–58. 28196787 10.1016/j.drugalcdep.2016.12.018

[c73] Smith J. L., Mattick R. P., Jamadar S. D., & Iredale J. M. (2014). Deficits in behavioural inhibition in substance abuse and addiction: A meta-analysis. Drug and Alcohol Dependence, 145, 1–33. 10.1016/j.drugalcdep.2014.08.009 25195081

[c74] Sobell L. C., & Sobell M. B. (1992). Timeline follow-back, A technique for assessing self-reported alcohol consumption In Litten R. Z. & Allen J. P. (Eds.), Measuring alcohol consumption, Psychosocial and biochemical methods (pp. 41–72). Totowa, NJ: Humana Press.

[c75] Stice E., Lawrence N. S., Kemps E., & Veling H. (2016). Training motor responses to food: A novel treatment for obesity targeting implicit processes. Clinical Psychology Review, 49, 16–27. 10.1016/j.cpr.2016.06.005 27498406

[c76] Suresh K. (2011). An overview of randomization techniques: An unbiased assessment of outcome in clinical research. Journal of Human Reproductive Sciences, 4, 8–11. 10.4103/0974-1208.82352 21772732 PMC3136079

[c77] Tiffany S. T., & Carter B. L. (1998). Is craving the source of compulsive drug use? Journal of Psychopharmacology, 12, 23–30. 10.1177/026988119801200104 9584965

[c78] Todd J., & Mullan B. (2014). The role of self-monitoring and response inhibition in improving sleep behaviours. International Journal of Behavioral Medicine, 21, 470–477. 10.1007/s12529-013-9328-8 23813124

[c79] Veling H., Aarts H., & Stroebe W. (2013). Stop signals decrease choices for palatable foods through decreased food evaluation. Frontiers in Psychology, 4, 875 10.3389/fpsyg.2013.00875 24324451 PMC3840792

[c80] Veling H., Holland R. W., & van Knippenberg A. (2008). When approach motivation and behavioral inhibition collide: Behavior regulation through stimulus devaluation. Journal of Experimental Social Psychology, 44, 1013–1019. 10.1016/j.jesp.2008.03.004

[c81] Veling H., van Koningsbruggen G. M., Aarts H., & Stroebe W. (2014). Targeting impulsive processes of eating behavior via the internet. Effects on body weight. Appetite, 78, 102–109. 10.1016/j.appet.2014.03.014 24675683

[c82] Verbruggen F., Best M., Bowditch W. A., Stevens T., & McLaren I. P. L. (2014). The inhibitory control reflex. Neuropsychologia, 65, 263–278. 10.1016/j.neuropsychologia.2014.08.014 25149820

[c83] Verbruggen F., & Logan G. D. (2008). Response inhibition in the stop-signal paradigm. Trends in Cognitive Sciences, 12, 418–424. 10.1016/j.tics.2008.07.005 18799345 PMC2709177

[c84] Verbruggen F., & Logan G. D. (2009). Models of response inhibition in the stop-signal and stop-change paradigms. Neuroscience and Biobehavioral Reviews, 33, 647–661. 10.1016/j.neubiorev.2008.08.014 18822313 PMC2696813

[c85] Verbruggen F., Logan G. D., & Stevens M. A. (2008). STOP-IT: Windows executable software for the stop-signal paradigm. Behavior Research Methods, 40, 479–483. 10.3758/BRM.40.2.479 18522058

[c86] Verbruggen F., McLaren I. P. L., & Chambers C. D. (2014). Banishing the control homunculi in studies of action control and behavior change. Perspectives on Psychological Science, 9, 497–524. 10.1177/1745691614526414 25419227 PMC4232338

[c87] Wallace P., Murray E., McCambridge J., Khadjesari Z., White I. R., Thompson S. G., . . . Linke S. (2011). On-line randomized controlled trial of an internet based psychologically enhanced intervention for people with hazardous alcohol consumption. PLoS ONE, 6(3), e14740 10.1371/journal.pone.0014740 21408060 PMC3052303

[c88] Weafer J., & Fillmore M. T. (2012). Alcohol-related stimuli reduce inhibitory control of behavior in drinkers. Psychopharmacology, 222, 489–498. 10.1007/s00213-012-2667-3 22358851 PMC4301262

[c89] Weafer J., & Fillmore M. T. (2015). Alcohol-related cues potentiate alcohol impairment of behavioral control in drinkers. Psychology of Addictive Behaviors, 29, 290–299. 10.1037/adb0000013 25134023 PMC4333136

[c90] Wiers R., Boffo M., & Field M. (in press). What’s in a trial? A conceptual framework for reviewing the effects of cognitive bias modification (CBM) in the treatment of addiction and a narrative review of the effects of CBM on alcohol use. Journal of Studies on Alcohol and Drugs.

[c91] Wiers R. W., Eberl C., Rinck M., Becker E. S., & Lindenmeyer J. (2011). Retraining automatic action tendencies changes alcoholic patients’ approach bias for alcohol and improves treatment outcome. Psychological Science, 22, 490–497. 10.1177/0956797611400615 21389338

