# Supplementary material for: A Randomized Controlled Trial of Inhibitory Control Training for the Reduction of Alcohol Consumption in Problem Drinkers
Source: J Consult Clin Psychol. 2018 Dec;86(12):991–1004. doi: 10.1037/ccp0000312 (PMC6277130; doi:10.1037/ccp0000312)
Supplement: Supplementary file 1 [file ICT_Online_Supplementary_CCP-2017-0854.docx]

**A randomised controlled trial of Inhibitory Control Training for the reduction of alcohol consumption in problem drinkers (supplementary materials)**

*Changes in alcohol consumption in the week before randomisation*

We investigated changes in alcohol consumption (in units per week) between the initial, baseline assessment (derived from the retrospective timeline follow-back drinking diary) and the self-monitoring phase (derived from the Down Your Drink drinking diary). A 2 (time; baseline, self-monitoring phase) x 4 (group: Associative No-Go, Associative Stop Signal, General, Control) mixed ANOVA demonstrated a significant main effect of time (*F*(1, 219) = 16.21, *p* < .01, *η_p_^2^* = .08), but no time x group interaction (*F*(3, 219) = 1.13, *p* = .34, *η_p_^2^* = .02). The main effect of time reflects a significant reduction in alcohol consumption from baseline to the monitoring phase (baseline = 80.60 ± 45.87 units, self-monitoring 70.31 ± 52.80 units).

*Stratification of participants based on reductions in alcohol consumption during the week of self-monitoring (Tables S1A and S1B)*

As detailed in the study protocol (Jones et al., 2014), our intention was to exclude ‘early reducers’ before conducting our primary analyses, because participants who drastically reduce their alcohol consumption after a brief intervention and a week of self-monitoring of alcohol consumption (Jenkins et al. 2009), may be less responsive to any additional interventions, thereby obscuring any effects of ICT (see Gual et al., 2013). In order to do this we followed a protocol for distinguishing early-reducers from non-reducers based on a previous RCT of nalmefene for a similar population to that studied here (Gual et al. 2013). Early reducers were classified as those participants who reduced their drinking to < 6 heavy drinking days per month *or* below the medium drinking risk level (EMEA 2014: average of 40g of alcohol per day if male, 20g if female) based on self-reports during the week of self-monitoring (before randomization). This stratification led to 53.2% of the overall sample being classified as early reducers. This was larger than our original estimate of 33% and would have required omission of data from more than half of the sample. Therefore, instead of our planned analyses we repeated all primary analyses with a further between subjects factor of early reducer status (early reducer vs. non early reducer). This does not reduce statistical power but does permit us to investigate if a large reduction in alcohol consumption before randomization could obscure (or indeed, maximise) any beneficial effects of ICT on drinking outcomes.

For total alcohol consumption, the pattern of results did not change, and there were no interactions involving group or reducer status (*F*s <1.14, *p*s >.34). Similarly, for heavy drinking days the pattern of results did not change, and there were no interactions involving group or reducer status (*F*s < 1.99, *p*s > .07).

*Participants’ motivation and ability to reduce alcohol consumption during the training phase*

During the post-training laboratory visit, participants were asked ‘*How motivated were you to reduce your alcohol consumption during the past month?*’ and ‘*How would you rate your ability to reduce your alcohol consumption during the past month?*’, both on a scale of 0-10. Overall motivation to reduce consumption during training was rated as 6.73 (± 1.97) and this was not significantly different across training groups (Associative No-Go (6.72 ± 1.68), Associative Stop Signal (7.18 ± 1.78), General (6.62 ± 2.05), Control (6.42 ± 2.95): *F* (3, 201) = 1.35, *p* = .26, *η_p_^2^* = .20). Overall ability was rated as 5.72 (± 2.11) and this was not significantly different across training groups (Associative No-Go (5.72 ± 1.97), Associative Stop Signal (5.41 ± 2.09), General (5.79 ± 2.19), Control (5.96 ± 2.12): *F* (3, 201) = 0.59, *p* = .62, *η_p_^2^* = .01).

*Secondary analyses – number of abstinent days (Table S2)*

The effect of training on the number of abstinent days was analysed using a 4 (group: Associative No-Go, Associative Stop Signal, General, Control) x 3 (time: baseline, after two weeks of training, after four weeks of training) mixed design ANOVA. The hypothesised group x time interaction was not significant and Bayes factors suggested support for the null hypothesis (*F*(6,402) = 0.20, *p* = .95, *η_p_^2^* = .014, BF^10^ = 0.01). There was a significant main effect of time (*F*(2, 402) = 98.76, *p* < .01, *η_p_^2^* = .33, BF^10^ > 99) and a significant main effect of group (*F*(3, 201) = 3.26, *p* = .02 , *η_p_^2^* =.05, BF^10^ = 1.66). Examination of the main effect of time demonstrated that the number of abstinent days increased from baseline to the first two weeks (*t*(204) = -11.28, *p* <.01, BF^10^ > 99) and was maintained (*t*(204) = -10.72, *p* < .01, BF^10^  > 99), but did not change (*t*(204) = 1.87, *p* = .09, BF^10^ = 0.43) from the first to the second two weeks of training. The main effect of group reflects the observation that, averaged across time, the associative Stop Signal ICT group had significantly fewer abstinent days than both the Associative No-Go training group (mean difference = 1.67, *p* =.07) and the Control group (mean difference= 1.70, *p* = .07). These group differences were present during the baseline assessment (before randomization), and were maintained during the ICT training period, and therefore they cannot be attributed to an effect of ICT.

*Individual differences in changes in proposed psychological mechanisms and their relationship to changes in drinking outcomes*

We examined whether individual differences in the change in proposed mechanisms of action of ICT (Inhibitory Control, Alcohol-Specific Inhibitory Control, and the D600 measure from the implicit association test) from baseline to post-test were associated with the reduction in alcohol consumption over the course of the training period. We computed change scores (Post-test minus Baseline) for each of these variables, and then ran multiple regression analyses to predict alcohol consumption (total volume of alcohol consumed) over the course of the 28-day training period. At step one we added alcohol consumption at baseline as a predictor, at step two we added baseline indices of candidate psychological mechanisms (SSRT, cue-specific SSRT, D600 score) and at step three we added the change scores. Across the whole sample the only significant predictor was alcohol consumption at baseline (*B* = .89 (95% CI .71 – 1.10); *p* < .01). This pattern of results did not change if groups were analysed separately. Therefore, individual differences in changes in candidate psychological mechanisms of action of ICT were not associated with changes in alcohol consumption over the course of the training period.

*Compliance and ‘dose’ (number of training sessions completed)*

Overall, participants who completed the minimum number of training sessions (8) completed 11.94 sessions on average (Associative No-Go = 11.91 (± 2.28), Associative Stop Signal = 11.61 (± 2.11), General = 11.87 (±1.88), Control = 12.36 (± 1.75)). There were no significant differences between groups (*F* (3, 201) = 1.18, p = .32, *η_p_^2^* = .017).

To examine whether compliance was related changes in alcohol consumption during the training period we conducted a multiple regression across the whole sample. Our outcome variable was alcohol consumption (total volume of alcohol consumed) over the course of the 28-day training period. At step one we added alcohol consumption at baseline as a predictor, and at step two we added the number of completed training sessions. The regression model was significant and predicted approximately 34% of the variance in alcohol consumption over the course of the training period (adjusted R^2^ = 0.34: *F*(2, 202) = 52.60, *p* < .01). Both baseline alcohol consumption (*B* = 0.88 (95% CI = 0.71 – 1.05), *p* < .01) and the number of sessions completed (*B* = -4.00 (95% CI -7.94 - -0.08), *p* = .02) were significant predictors of alcohol consumption during training. This demonstrates that the more training sessions that participants completed, the more their alcohol consumption reduced over the course of training. However, when we repeated these analyses separately for each group, the number of sessions completed was not a significant predictor of alcohol consumption in any group (ps > .24), an effect that is likely to reflect reduced statistical power when groups are analysed separately.

*Training performance: progression in associative Stop Signal and General training*

In the associative Stop Signal and general Stop Signal training groups the SSD was dependent on previous training performance: If participants successfully inhibited on more than 50% of stop signal trials in a given training session, the SSD increased by 10ms in the subsequent training session, therefore making inhibition more difficult. For both tasks, participants consistently inhibited on more than 50% of trials so SSD increased after almost every training session. In the Associative group, SSD increased on average 10.51 (± 2.42) times (average number of sessions completed = 11.62 (± 2.09)), whereas in the general group the SSD increased 11.15 ± (1.89) times (average number of sessions completed = 11.87 (±1.88)). The level of progression did not significantly differ between groups (*t* (99) = 1.49, *p* = .139).

To examine whether progression was related changes in alcohol consumption during the training period we conducted a multiple regression across the associative Stop Signal and General training groups. Our outcome variable was alcohol consumption (total volume of alcohol consumed) over the course of the 28-day training period. At step one we added alcohol consumption at baseline as a predictor, and at step two we added the number of final SSD reached. The regression model was significant and predicted approximately 47% of the variance in alcohol consumption over the course of the training period (adjusted R^2^ = 0.47: *F*(2, 100) = 44.94, *p* < .01). Baseline consumption was a significant predictor of alcohol consumption during training (*B* = 1.14 (95% CI .90 – 1.37); p < .01), however final SSD was not a significant predictor (B = -3.92 (95% CI -9.00 – 1.17); p = .13). Analysing each group separately did not change the pattern of results. Similar findings were also demonstrated for heavy drinking days and abstinent days.

*Training performance: successful inhibition during training*

A recent meta-analysis (Jones et al., 2016) demonstrated a significant association between successful inhibition to appetitive cues during training and the magnitude of the effect of ICT on drinking outcomes in the laboratory. There was a significant difference between the proportion of correct inhibitions to alcohol-related cues in the Associative No-Go (98.64% ± 1.37) and Stop Signal groups (87.38% ± 11.54; t (101) = 7.12, p < .01). This is is almost certainly a consequence of the differing inhibitory pressures in the different tasks, but also because inhibition became more difficult in the Stop Signal group (but this remain consistent across training sessions in the No-Go group).

To examine if the proportion^[[1]](#footnote-1)^ (expressed as an overall percentage, across all completed training sessions) of successful inhibition-alcohol trials had an influence on alcohol consumption during training we conducted a multiple regression. Our outcome variable was alcohol consumption (total volume of alcohol consumed) over the course of the 28-day training period. At step one we added alcohol consumption at baseline as a predictor, and at step two we added the proportion of successful inhibition-alcohol trials. The overall regression model was significant and explained approximately 27% of variance (adjusted R^2^ = 0.27, F(1,100) = 19.59, *p* < .01). Baseline alcohol consumption was a significant predictor (B = .93 (95% CI 0.63 – 1.23), *p* < .01), however proportion of successful inhibition trials was not (B = -.70 (95% CI -1.93 - .54), *p* = .226). Analysing each group separately did not influence our results. Similar findings were also demonstrated for heavy drinking days and abstinent days.

*Follow-up analyses: heavy drinking and abstinent days (Table S3)*

We analysed differences in heavy drinking days at follow up using a 4 (group: Associative No-Go, Associative Stop Signal, General, Control) x 3 (time: two week, four week, six week follow up) ANOVA. There was no main effect of time (F(2, 154) = 2.84, p = .06, *η_p_^2^* = .04) or group x time interaction (F(6,154) = 0.67, p = .67, *η_p_^2^* = .03). For abstinent days the same pattern of results was observed, with no main effect of time (F(2, 154) = 0.94, p = .39, *η_p_^2^* = .01) or time x group interaction (F(6, 154) = 0.60, p = .73, *η_p_^2^* = .02).

*Follow-up analyses: accounting for missing data*

As there was a substantial amount of missing follow-up data across the three follow-ups we also repeated analyses with the last observation carried forward (LOCF). If participants did not provide any data at 2-week follow-up the last observation carried forward was derived from the final 2 weeks of training. A 4 (group: Associative No-Go, Associative Stop Signal, General, Control) x 3 (time: two week, four week, six week follow up) ANOVA for heavy drinking days demonstrated no significant main effect of time (F(2, 402) = 2.98, p = .05, *η_p_^2^* = .015) or time x group interaction (F(6, 402) = 0.27, p = .95, *η_p_^2^* = .004). A similar pattern of results was demonstrated for abstinent days (main effect of time: (F(2, 402) = 0.89. p = .413, *η_p_^2^* = .004; time x group interaction: (F(6, 402) = 1.44, p = .200, *η_p_^2^* = .021).

*Thematic analyses of participant feedback (Table S4)*

We conducted a thematic analysis of transcripts of exit interviews to identify any themes related to accessibility, usage and experiences of using the application. Two authors (AJ and ER) independently examined transcripts and identified themes. Any minor disagreements were resolved through discussion We identified four potential themes, three around accessibility and engagement, and one around effectiveness.

*Accessibility*

The medium in which training was presented was highlighted as important. Participants suggested that to improve accessibility it could be administered on portable devices such as tablets and mobile phones as an ‘app’. Furthermore, some participants suggested that text alerts, rather than email would improve compliance.

*Engagement*

Participants reported that the training tasks failed to engage their attention throughout the four week period, and having the same task with little variety was described as ‘monotonous’, ‘predictable’ and ‘boring’. Few suggestions were made to improve the engagement. The most common was to ‘*gamify*’ the training by using animation or making it more interactive. Other suggestions included personalization, and increasing the variation of images and tasks.

*Understanding*

A lack of understanding about the intended purpose of the training was highlighted as an issue with participants. They expressed a desire for more information about the aim of the training tasks prior to undertaking the training. Because of this some participants were sceptical about its likely effectiveness and whether it would help them to drink less.

*Measuring progress*

Task feedback was considered useful by the participants, with some suggesting it motivated them to try harder. The ability to track data and make comparisons was highlighted as a potential improvement in order to monitor progress over time.

*Table S1. Alcohol consumption (in UK units per fortnight) (Panel A) and Heavy Drinking days (Panel B) across time points, groups and early reducer status. Values are Means (Standard Deviations)*

**A. Alcohol consumption** **Control Go/No-Go Cue-Specific General**

**Early Reducers**

Baseline 71.04 (33.90) 61.06 (38.80) 58.35 (21.28) 67.27 (30.01)

First 2 weeks training 41.10 (24.27) 40.86 (37.27) 36.23 (20.87) 41.99 (24.98)

Second 2 weeks training 42.43 (22.28) 34.84 (23.99) 40.79 (18.37) 39.25 (24.25)

**Non early-reducers**

Baseline 93.51 (63.97) 91.88 (34.82) 104.02 (48.17) 105.52 (60.33)

First 2 weeks training 58.01 (33.21) 58.63 (35.85) 86.12 (45.99) 70.04 (53.21)

Second 2 weeks training 58.15 (38.19) 67.48 (46.62) 86.20 (32.48) 67.40 (51.58)

**B. Heavy drinking days** **Control Go/No-Go Cue-Specific Genera l**

**Early reducers**

Baseline 5.00 (3.16) 4.14 (3.40) 3.82 (2.48) 4.96 (3.28)

First 2 weeks training 2.63 (2.30) 2.03 (1.82) 2.50 (1.99) 2.61 (1.55)

Second 2 weeks training 2.79 (1.82) 2.03 (1.95) 2.71 (1.80) 2.07 (1.49)

**Non early-reducers**

Baseline 5.73 (3.85) 6.88 (3.97) 6.95 (4.06) 7.65 (3.51)

First 2 weeks training 3.85 (2.59) 4.04 (2.95) 5.62 (3.89) 4.54 (3.67)

Second 2 weeks training 3.27 (2.27) 4.24 (2.93) 5.95 (3.40) 4.41 (3.51)

*Table S2. Mean number of abstinent days per fortnight across time points and groups. Values are Means (Standard Deviations)*

**Control Go/No-Go Cue-Specific Genera l**

Baseline 5.65 (3.21) 5.76 (3.95) 3.95 (3.30) 5.10 (3.38)

First 2 weeks training 8.46 (3.45) 8.24 (3.65) 6.92 (3.68) 7.69 (3.90)

Second 2 weeks training 8.16 (3.37) 8.12 (3.60) 6.29 (3.18) 7.50 (3.98)

*Table S3: Secondary outcomes of follow-up data. Values are Means (Standard Deviations)*

Control Go-No Cue-Specific General

**Heavy Drinking days**

First 2 weeks 2.72 (2.12) 3.08 (2.29) 3.18 (3.14) 2.90 (2.81)

*LOCF 2.72 (2.12) 2.69 (2.40) 3.37 (3.26) 3.00 (2.84)*

Second 2 weeks 2.86 (1.93) 3.32 (2.67) 3.10 (2.68) 3.08 (1.89)

*LOCF 2.84 (1.99) 2.78 (2.60) 3.24 (3.07) 3.23 (2.65)*

Third 2 weeks 3.17 (2.69) 3.32 (2.07) 3.32 (2.88) 3.63 (3.73)

*LOCF 2.98 (2.34) 2.89 (2.61) 3.71 (3.13) 3.48 (3.23)*

**Abstinent days**

First 2 weeks 9.27 (3.16) 8.82 (3.33) 7.03 (4.11) 7.90 (4.17)

*LOCF 8.86 (3.22) 8.61 (3.50) 7.14 (3.94) 7.73 (4.24)*

Second 2 weeks 8.86 (3.12) 8.27 (3.72) 8.13 (3.81) 8.78 (3.68)

*LOCF 8.92 (3.11) 8.37 (3.75) 8.08 (3.88) 7.83 (4.35)*

Third 2 weeks 8.54 (3.42) 8.89 (3.13) 8.92 (3.37) 8.54 (3.41)

*LOCF 8.86 (3.16) 8.30 (3.78) 7.86 (3.96) 7.81 (4.42)*

*Legend: LOCF = Last observation carried forward estimate.*

*Table S4: Thematic analysis from post-intervention interview: Themes and example quotes from participants*

**Theme 1: Accessibility**

Ppt.91 ‘It would be easier if it was on a tablet’

Ppt.194 ‘Tablets and stuff are a lot more accessible than laptops or PCs.’

**Theme 2: Engagement and how to improve**

Ppt.152 ‘ I thought the tasks were quite boring’

Ppt.201 ‘It was very cold, maybe personalise it’.

Ppt.208 ‘I think I might have missed some but I don’t remember, probably because they were so boring’

Ppt.213 ‘It would have to be a lot more interesting. If you could make it more like a game’

**Theme 3: Understanding**

Ppt. 156 **‘**The tasks, again because I didn’t understand them, I didn’t think they were effective’

Ppt. 177 ‘It was hard to understand what the goal was, in terms of whether it was trying to put you off by looking at them … I don’t know’

Ppt. 215 ‘I’m not sure how they would work or make me drink less’

**Theme 4: Measuring progress**

Ppt. 88’ I thought it was good the way you got the percentages at the end so it was sort of a little test and you know you could try to improve on each occasion’

Ppt. 160 ‘If you could track the data to see where you compared from one week to the next it would something to measure your progress’

Ppt.208 ‘interesting to see and gauge the change in reactions’

1. Proportions were used rather than raw scores as there were 100 alcohol-inhibition trials in the Associative No-Go training sessions, and 50 in the Associative Stop Signal sessions. [↑](#footnote-ref-1)
